# Exercise interventions and serum IGF-1 levels in older adults with frailty and/or sarcopenia: a systematic review and meta analysis

**DOI:** 10.3389/fpubh.2025.1660694

**Published:** 2025-08-21

**Authors:** Rui Chu, Mingming Li, Yeshou Xie, Yinuo Du, Tao Ni

**Affiliations:** ^1^Anhui Polytechnic University, Wuhu, Anhui, China; ^2^Hefei Preschool Education College, Hefei, Anhui, China; ^3^Changzhou University, Changzhou, Jiangsu, China

**Keywords:** sarcopenia, frailty, insulin-like growth factor-1, exercise intervention, meta-analysis

## Abstract

**Objective:**

Insulin-like growth factor-1 (IGF-1) is thought to play an important role in regulating skeletal muscle mass and function, with its decline potentially linked to age-related frailty and sarcopenia. Given the limitations of pharmacological and nutritional interventions, exercise may serve as a potential non-pharmacological strategy to modulate IGF-1 levels. The purpose of this study is to systematically evaluates the effects of exercise interventions on serum IGF-1 levels in older adults with frailty and/or sarcopenia using a meta-analysis approach.

**Methods:**

A systematic search was conducted in PubMed, Web of Science, Cochrane Library, EMBASE and Scopus (from inception to July 2025) to identify randomized controlled trials (RCTs) investigating the impact of exercise interventions on serum IGF-1 levels in older adults with frailty and/or sarcopenia. Data were analyzed using RevMan 5.4 and Stata 15.1, with standardized mean differences (SMD) and 95% confidence intervals (95% CI) calculated via a random-effects model. The protocol was registered with PROSPERO (CRD420251085472).

**Results:**

A total of 11 studies (comprising 16 RCTs) were included, involving 604 participants (intervention group: 314; control group: 290), age range: 63.6 to 85.8 years old. Meta-analysis revealed that exercise interventions significantly increased serum IGF-1 levels in older adults with frailty and/or sarcopenia (SMD = 0.42, 95% CI: 0.23–0.60, *p* < 0.0001, *I*^2^ = 15%). Subgroup analysis demonstrated that combined training (aerobic + resistance) yielded the most pronounced effect (SMD = 0.60, 95% CI: 0.36–0.84, *p* < 0.00001, *I*^2^ = 0%), followed by resistance training alone (SMD = 0.35, 95% CI: 0.05–0.66, *p* = 0.02, *I*^2^ = 28%), whereas aerobic training alone showed no significant effect [SMD = 0.01, 95%CI: (−0.46, 0.48), *p* = 0.96, *I*^2^ = 0%]. Similarly, subgroup analysis revealed that exercise intervention could effectively improve serum IGF-1 levels in older adult individuals with frailty (SMD = 0.53, 95%CI: 0.07–0.98, *I*^2^ = 0%) or sarcopenia (SMD = 0.40, 95%CI: 0.19–0.61, *I*^2^ = 25%), with no statistically significant difference in effect sizes between the two groups.

**Conclusion:**

Exercise intervention can effectively increase serum IGF-1 concentrations in older adults with frailty and/or sarcopenia. The research results may provide key evidence-based basis for clinical non-pharmacological interventions.

**Systematic review registration:**

https://www.crd.york.ac.uk/PROSPERO/view/CRD420251085472.

## 1 Introduction

Sarcopenia is a geriatric syndrome characterized by progressive and generalized decline in muscle strength and mass, along with impaired balance ability, which increases the risk of falls, physical disability, mortality, and other adverse outcomes (1). This condition exhibits clinical heterogeneity and frequently coexists with metabolic disorders such as osteoporosis and obesity, leading to complications including osteosarcopenia, sarcopenic obesity, and osteosarcopenic obesity (2–4). Frailty, on the other hand, is defined by a decline in physiological reserve, impaired stress response, and disrupted homeostasis in older adults, often presenting with multisystem dysfunction such as reduced mobility, weight loss, and cognitive impairment (5, 6). Sarcopenia is considered an early manifestation of frailty, with shared pathological mechanisms including chronic low-grade inflammation, hormonal dysregulation, malnutrition, and sedentary behavior (7). Both conditions are clinically characterized by functional decline, particularly in balance impairment, muscle function, and strength reduction (7). Against the backdrop of global population aging, the prevalence of sarcopenia and frailty continues to rise (2, 8–10), and both are strongly associated with adverse health outcomes such as falls, hospitalization, chronic disease progression, and all-cause mortality (11–13). Given their profound impact on geriatric health and healthcare burdens, effective prevention and management of frailty and sarcopenia have become critical medical priorities for improving healthy longevity and optimizing healthcare resource allocation.

Insulin-like growth factor-1 (IGF-1) is a pleiotropic polypeptide hormone structurally similar to insulin. It is mainly synthesized in the liver and secreted into the bloodstream (endocrine effect), regulated by growth hormone (GH). Meanwhile, peripheral tissues such as skeletal muscle can also locally synthesize and secrete IGF-1, exerting autocrine/paracrine effects (14). IGF-1 is a key factor regulating the anabolic and catabolic balance of skeletal muscle and determining muscle size and function (15). After the age of 21, serum IGF-1 concentrations in humans show a progressive downward trend with increasing age, and this decline is closely associated with the onset and progression of sarcopenia (16). The reason why maintaining IGF-1 levels is effective in preventing and delaying sarcopenia lies in its dual regulatory role in skeletal muscle protein metabolism: IGF-1 binds to IGF-1 receptors (IGF-1R) on muscle cell surfaces, activating multiple intracellular signaling cascades (such as the PI3K/Akt/mTOR pathway, PI3K/Akt/GSK3β pathway, and MAPK/ERK pathway), thereby promoting muscle protein synthesis (17, 18). At the same time, IGF-1 can also “turn off” the expression of muscle atrophy genes at the transcriptional level through signal transduction via its PI3K/Akt/FoxO pathway, thereby inhibiting protein degradation (19–21). A Mendelian randomization study based on genetic evidence further suggests a causal association between genetically predicted higher IGF-1 levels and lower sarcopenia risk (22). In addition, the relationship between IGF-1 and sarcopenia/frailty is not a one-way causal chain but a complex interaction network. Pathological states themselves can in turn profoundly affect the function of the IGF-1 system. Studies have shown that factors such as malnutrition, chronic low-grade inflammation, and reduced physical activity in the context of sarcopenia may further inhibit the synthesis and secretion of IGF-1 in the liver and muscles (23); in local tissues, hydrolytic cleavage of IGF-binding proteins (IGFBPs) is a key step in releasing free IGF-1 and regulating its bioavailability (24), but this regulatory process may be disrupted in the pathological microenvironment of sarcopenia, thereby inhibiting IGF-1 signaling (25).

While pharmacological interventions (e.g., recombinant human growth hormone [rhGH], somatostatin analogs) and nutritional strategies (e.g., high-protein diets, amino acid supplementation) can effectively elevate serum IGF-1 levels, they are limited by poor adherence, high costs, and potential side effects (26–28). Compared with drug and nutritional therapies, exercise training is characterized by greater safety and more cost-effective alternative. However, its controversy in the effect of increasing serum IGF-1 concentrations has attracted much attention, with the controversy focusing on whether exercise intervention can effectively increase serum IGF-1 concentrations. Some studies have shown that endurance and resistance training can promote the release of inflammatory cytokines and growth factors including IGF-1 by inducing remodeling of muscle ultrastructure, suggesting that exercise may have the potential to increase serum IGF-1 (29, 30). In contrast, other studies have found that serum IGF-1 concentrations do not increase significantly or even decrease following either acute or long-term exercise interventions, which is in clear disagreement with the aforementioned conclusions (31, 32).

Additionally, individual randomized controlled trials (RCT) often suffer from small sample sizes and heterogeneous intervention protocols. Existing studies have conducted comprehensive quantitative analyses of results from multiple randomized controlled trials through meta-analysis. For instance, Titus et al., Behrad et al., and Jiang et al. all used meta-analytic methods to, respectively, verify the improvement effect of exercise intervention on serum IGF-1 concentration in healthy middle-aged and older adult individuals (33–35). However, none have specifically focused on older adults with frailty and/or sarcopenia, limiting their clinical applicability. These inconsistencies highlight the need for more systematic investigations to clarify the effects of exercise on IGF-1 in this population.

To address these gaps, based on the proposed hypothesis (exercise interventions, particularly those combining resistance and aerobic training, will effectively elevate serum IGF-1 levels in older adults specifically diagnosed with frailty and/or sarcopenia), this study employs meta-analysis to quantitatively synthesize existing RCT evidence on the effects of exercise interventions on serum IGF-1 levels in older adults with frailty and/or sarcopenia. Through systematic review, we further elucidate the underlying mechanisms by which different exercise modalities may enhance IGF-1 production. Additionally, subgroup analyses are conducted to compare the efficacy of various exercise types. By integrating these approaches, this study aims to address existing research gaps by validating the effects of exercise interventions on serum IGF-1 levels in these patients, thereby providing comprehensive theoretical foundations and practical guidance for the prevention and treatment of related conditions, as well as the development of clinical trials.

## 2 Methods

This study was conducted in accordance with the PRISMA (Preferred Reporting Items for Systematic Reviews and Meta-Analyses) guidelines for systematic reviews and meta-analyses (36). The protocol has been registered in the PROSPERO database of the Centre for Reviews and Dissemination at the University of York: registration number CRD420251085472.^1^

### 2.1 Search strategy

The articles search process involved independent screening by multiple reviewers (MML and YND). The retrieval time span covers from the establishment of each database to July 20, 2025, and the retrieval scope includes electronic databases such as Web of Science, Cochrane Library, PubMed, EMBASE, Scopus, and other relevant electronic databases. The search strategy adopted a combination of subject terms and free terms, covering the following concept groups: (1) Terms related to IGF-1; (2) Terms related to exercise intervention; (3) Terms related to the target population (older adults + frailty/sarcopenia). A detailed list of specific search terms and Search Strategy is provided in Tables 1, 2.

**Table 1:** List of specific search terms.

Concept groups	Search terms
Insulin like growth factor-1	Insulin-Like Growth Factor I, IGF-1, Insulin Like Growth Factor I, Somatomedin C
Exercise intervention	Exercise, Physical Exercise, Aerobic Exercise, Blood Flow Restriction Therapy, High-Intensity Interval Training, Resistance Training, Endurance Training
Target population	senior citizen, Aged, Older adult, Frailty, Sarcopenia, Frailties, Frailness, Frailty Syndrome, Sarcopenias
Research type	Randomized controlled trial

**Table 2:** List of search syntax.

Search Strategy	Search terms
#1	Exercise/ OR Physical Exercise/OR Aerobic Exercise/OR Blood Flow Restriction Therapy/OR High-Intensity Interval Training/OR Resistance Training/OR Endurance Training/
#2	Insulin-Like Growth Factor I/OR IGF-1/OR Insulin Like Growth Factor I/OR Somatomedin C/
#3	Senior citizen/OR Aged/ OR Older adult/
#4	Frailty/OR Frailties/OR Frailness/OR Frailty Syndrome
#5	Sarcopenia/OR Sarcopenias/
#6	Randomized controlled trial
#7	#4 OR #5
#8	#1 AND #2 AND #3 AND #6 AND #7

After the retrieval, duplicate articles were first removed by EndNote articles management software. Then, according to the pre-established inclusion and exclusion criteria, the titles and abstracts of the articles were initially screened to exclude obviously irrelevant studies. For the articles that passed the initial screening, the full texts were further obtained, and two independent researchers (MML and YND) conducted secondary screening, quality evaluation and data extraction, respectively. In case of disputes, the corresponding author organized a discussion meeting to negotiate and resolve them.

### 2.2 Inclusion and exclusion criteria

Based on the PICOS principle of Cochrane, the inclusion and exclusion criteria for articles formulated in this study are as follows:

#### 2.2.1 Inclusion criteria

(1) Study subjects: older adult individuals aged ≥60 years (regardless of gender) from all settings (including communities, nursing homes, hospitals, etc.) who met the clinical diagnostic criteria for sarcopenia and/or frailty were included. Among them, the diagnostic criteria adopted for sarcopenia are as follows: EWGSOP2 (2010): grip strength: male < 27 kg, female < 16 kg; DXA: male < 7.0 kg/m^2^, female < 5.5 kg/m^2^; BIA: male < 7.0 kg/m^2^; female < 5.7 kg/m^2^, gait speed ≤ 0.8 m/s (37); AWGS (2019): grip strength: male < 28 kg, female < 18 kg; gait speed: < 1.0 m/s; muscle mass: DXA male < 7.0 kg/m^2^, female < 5.4 kg/m^2^ (38). The diagnostic indicators for older adult frailty are as follows: Frailty Phenotype (2001): among the five criteria of unintentional weight loss, self-reported fatigue, low grip strength, slow gait speed, and insufficient physical activity, ≥3 criteria indicate frailty; 1–2 criteria indicate pre-frailty; 0 criteria indicate health (39); Frailty Index: the proportion is calculated based on more than 30 health deficits (symptoms, diseases, dysfunctions), and a Frailty Index (FI) ≥ 0.25 is used to determine frailty (40); Clinical Frailty Scale, CFS (2005): the frailty degree of the older adult is quantitatively evaluated through scoring in terms of their self-perceived ability, walking ability, cognitive ability, emotional state, etc. The CFS scale is usually divided into 9 levels, ranging from very healthy to end-stage, among which levels 1–3 are defined as healthy, levels 4–5 as intermediate frailty, and levels 6 and above as frailty) (41). (2) The experimental group received interventions using single or multiple exercise modalities, including aerobic training (such as brisk walking, stationary cycling, running, balance training, calisthenics, etc.), resistance training (including resistance exercises performed with various equipment), and combined training (conducting both aerobic and resistance training in the same training session). Among these, the exercise interventions were required to be continuous periodic interventions with a duration of at least 4 weeks and a frequency of 2–5 times per week. (3) Control measures: The control group adopts measures such as health education, routine nursing, and routine exercise (Maintain one’s original daily living activities), which were different from the experimental group in terms of intervention methods or intervention objects. (4) Study type: Only randomized controlled trials (RCTs) are included. (5) Outcome indicators: The outcome indicators involved in the study should include Serum IGF-1 Levels^(ng/ml)^. (6) Language restriction: To cover all existing relevant studies as comprehensively as possible, this study does not impose restrictions on the language of the articles included.

#### 2.2.2 Exclusion criteria

(1) Non-randomized controlled trial studies. (2) The intervention measures in the experimental group are inconsistent, such as using other drug interventions or failing to clearly define the type of exercise. (3) The study subjects do not meet the requirements, including non-human subjects, the average age of the intervention objects is under 60 years old, or the diagnostic criteria or guidelines for frailty and sarcopenia are not mentioned. (4) The study data are incomplete and cannot be supplemented. (5) The study results do not report IGF-1 data or the data cannot be extracted. (6) Grey literature: including unpublished articles, conference papers, dissertations, theses, and other similar materials.

### 2.3 Data extraction

Two reviewers (MML and YND) independently extracted data. If there were disagreements during the extraction process, the corresponding author would organize a meeting to negotiate and resolve them. The specific contents of the extracted data are as follows:

1. Basic information of the articles: first author and year of publication.
2. Subject information: subject characteristics such as sample size, age, physical status (frailty and/or sarcopenia) and diagnostic criteria.
3. Intervention parameters: Specific implementation details of the intervention measures adopted in the experimental group were extracted, and information on intervention parameters such as intervention duration, frequency, and intensity was analyzed according to research needs.
4. Experimental result data: for the intervention results of IGF-1, the final values of the experimental group and the control group after intervention were extracted, and the data were presented in the form of mean ± standard deviation (mean ± SD).

### 2.4 Quality assessment

Risk of bias was assessed using the Cochrane Risk of Bias Tool (RoB2) (42), conducted independently by two reviewers (MML and YND). Disagreements were resolved through discussion with the corresponding author. The following domains were evaluated:

1. Selection bias (random sequence generation): It is necessary to judge whether the allocation of research subjects has truly achieved randomness, and whether the allocation scheme has been effectively concealed before the completion of allocation, so as to prevent researchers or subjects from predicting the grouping situation and thus avoiding selection bias.
2. Performance bias (deviations from intended interventions): Assess whether the trial successfully implemented blinding for participants and personnel delivering the intervention (such as doctors and nurses). If blinding was not achieved, determine whether there were systematic deviations in the intervention that might affect the outcomes.
3. Attrition bias (incomplete outcome data): Judge whether the outcome data are complete. If incomplete, determine whether the proportion and reasons for data missing may be related to the true outcome values, thereby leading to bias in the effect estimates between groups.
4. Detection bias (inappropriate outcome measurement): Judge whether the measurement methods of outcomes are objective and reliable, and whether outcome assessors remain blinded to participants’ grouping information to prevent subjective judgments from being influenced by grouping information.
5. Reporting bias (selective reporting of results): Judge whether the research results presented in the final report are derived from pre-specified research protocols and analysis plans, or are presented after “data mining” or “data cherry-picking.”
6. Other potential sources of bias: It logically integrates the assessment results of the previous five core domains rather than being an independent source of bias. The overall risk of bias is judged as “high risk” if at least one of the five core domains is rated “high risk”; with no domain rated “high risk,” it is “some concerns” if at least one domain is rated so; and it can only be “low risk” when all five core domains are rated “low risk.”

Each domain was rated as “low risk,” “high risk,” or “unclear risk.” Based on the overall quality, studies were categorized into three grades:

- Grade A: All domains rated as low risk.
- Grade B: Some domains rated as low risk.
- Grade C: No domains rated as low risk.

### 2.5 Statistical analysis

In this study, RevMan 5.4 and Stata 15.1 software were used to conduct a meta-analysis on the outcome indicators of the included articles, aiming to clarify the intervention effect of exercise intervention on IGF-1 in older adults with frailty and/or sarcopenia. Considering that all outcome indicators of the included articles are continuous variables with inconsistent measurement units, the standardized mean difference (SMD) and its 95% confidence interval (CI) were used for statistical analysis.

In terms of heterogeneity assessment, the *I*^2^ statistic and Q test were used for judgment: when *I*^2^ ≤ 50% and *p* ≥ 0.01, it was judged as low heterogeneity; when *I*^2^ > 50% and *p* < 0.01, it was judged as high heterogeneity. In cases of high heterogeneity, subgroup analysis and meta-regression analysis were employed to identify the sources of heterogeneity. Additionally, sensitivity analysis and publication bias detection were conducted to analyze the reliability of the research results. To evaluate potential publication bias, Egger’s test was used for statistical analysis in this study; if publication bias was detected, the trim-and-fill method was used to assess the stability of the overall effect of the study. In addition, sensitivity analysis was performed by excluding the included articles one by one to test the robustness of the meta-analysis results. Meanwhile, due to differences in the biological mechanisms of IGF-1 affected by different types of exercise interventions (such as aerobic training, resistance training, and comprehensive training, etc.), different intervention effects may occur. Therefore, subgroup analysis will be conducted to explore the differences in the impact of different exercise intervention types on outcome indicators.

## 3 Results

### 3.1 Studies search results

**Figure 1:**
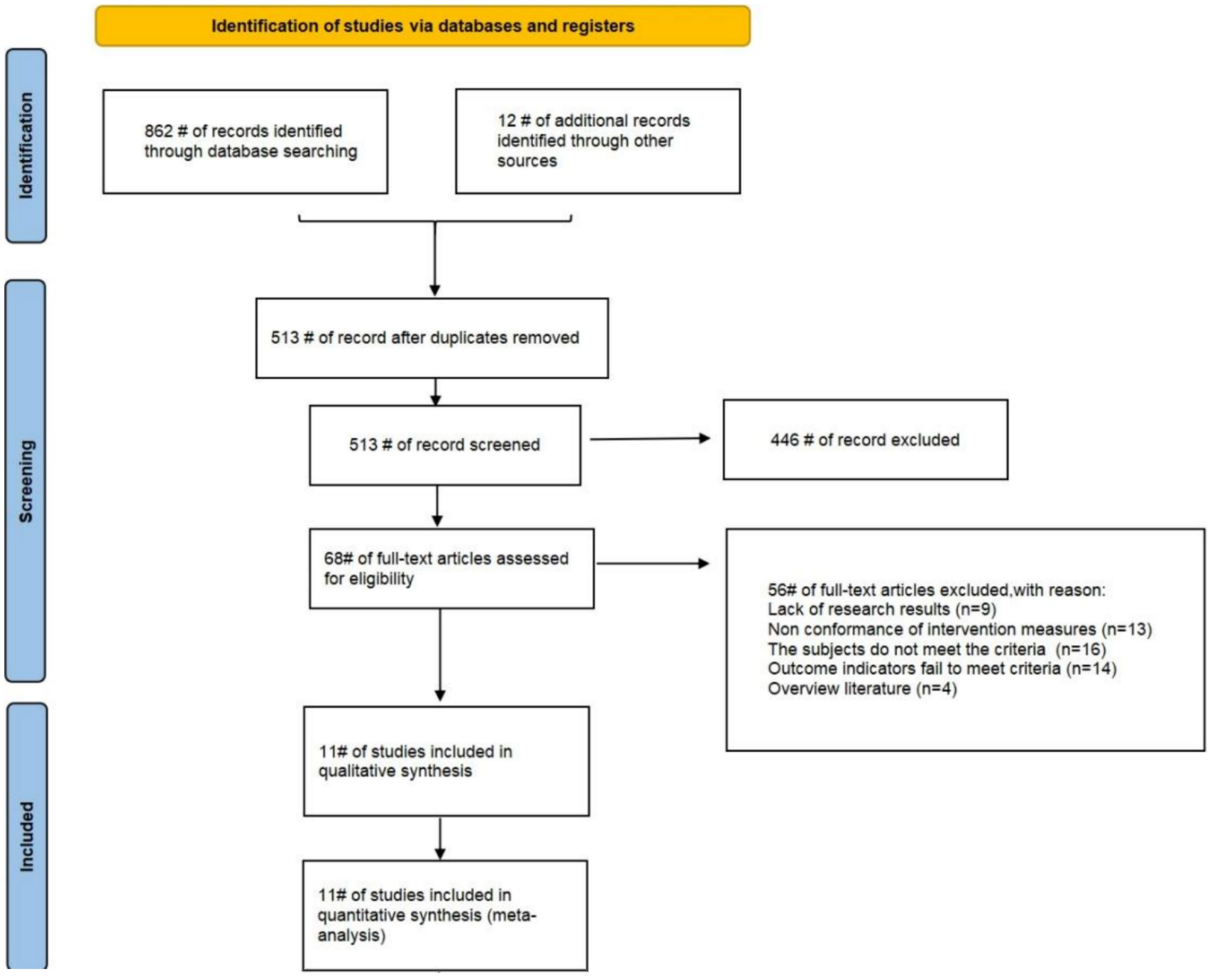
Studies selection process.

This study comprehensively searched multiple electronic databases and initially identified 874 relevant articles. First, EndNote was used for duplicate checking, 361 duplicate articles were excluded, leaving 513 valid articles. Then, preliminary screening was conducted by reading titles and abstracts, and 446 irrelevant articles were excluded based on research topics and keywords. Finally, the remaining 67 articles were evaluated through full-text intensive reading in accordance with the pre-established inclusion and exclusion criteria, and 11 eligible articles were ultimately included. Among them, since some articles adopted different exercise intervention measures or exercise intervention frequencies in multiple groups, 16 RCTs were included in these 11 articles. The studies screening process is shown in Figure 1.

### 3.2 Characteristics of the studies

Table 3 systematically summarizes the subject characteristics, experimental grouping design, exercise intervention protocols, and related outcome indicators of the 11 included articles (including 16 RCTs) (43–53). Among them, the total sample size is 604 cases, including 314 cases in the experimental group and 290 cases in the control group. The average age of all participants in the experiment is approximately 71.8 years old.

**Table 3:** Characteristics of included studies.

Study	Sample size (ETG/CG)	Age (ETG/CG)	Subject Characteristics	Intervention				Control group	Outcome
				Mode	Training movement	Intensity	Duration days/week (weeks)		
Rondanelli et al. (2016) (43)	ETG:69 CG:61	ETG:80.77 ± 6.29 CG:80.21 ± 8.54	Sarcopenia	AT+RT	AT: Single-leg standing, multi-directional left of gravity transfer, tandem standing, tandem walking, etc. RT: Ankle weighting for knee flexion and extension in sitting and standing positions; elastic band resistance training for upper and lower limbs.	Borg:12–14	5(12)	Nutritional supplementation	IGF-1
Urso et al. (2005) (44)	ETG:5 CG:5	ETG:85.8 ± 3.63	Frail older adult	RT	RT: Resistance training for the extensor muscles of the lower extremity hips and knees using pneumatic resistance training equipment.	50% 1-RM	3(10)	Non-exercise	IGF-1
Huovinen et al. (2016) (45)	ETG:37 CG:11	ETG:71.9 ± 3.1 CG:71.8 ± 2.9	Sarcopenia	RT	RT: Multiple resistance training for major muscle groups of the upper and lower limbs, including leg press, chest press, seated rowing, sit-up, back extension, seated leg curl, and hip abduction.	50–80% 1-RM	3(16)	Non-exercise	IGF-1
Park et al. (2021) (46)	ETG:10 CG:9	ETG:66.6 ± 3.98 CG:64.8 ± 3.80	Sarcopenia	AT+RT	AT: Marching on the spot combined with wide shoulder abduction/adduction, alternating touch-down walking, core stability and balance exercises, single-leg standing, etc. RT: Supine pelvic tilt, half-crunch, alternating knee extension, kneeling bird-dog, plank, etc.	50–60% HRR	5(15)	patients with osteoarthritis	IGF-1
Hooshmandi et al. (2024) (47)	ETG(1):10 ETG(2):10 CG:10	ETG(1):64.80 ± 3.01 ETG(2):67.8 ± 3.79 CG:65.40 ± 2.27	Sarcopenia	RT	RT (1): During the first six weeks of training, the “rest-pause” technique was adopted. Participants first completed 4–6 repetitions of HIIRT movements, rested for 20 s, and then performed two more repetitions until exhaustion. RT (2): In the training of the last four weeks, the experimental group was divided into two subgroups, which conducted HIIRT training with half and one-third of the initial training volume, respectively.	ETG(1): 60–85% 1-RM; ETG(2): 50% 1-RM、 30% 1-RM.	2–3(10)	Non-exercise	IGF-1
Santos et al. (2024) (48)	ETG:16 CG:18	ETG:69.3 ± 7.4 CG:68.4 ± 6.9	Sarcopenia	RT	RT program for both groups composed of 8 exercises performed in the following order: chest press, horizontal leg press,seated row, leg extension, preacher curl, leg curl, triceps push-down, and seated calf raise, each exercise was performed in 1set of 10–15 repetitions	60% 1-RM	3(12)	low-frequency exercise	IGF-1
Zhuang et al. (2025) (49)	ETG:13 CG:14	ETG:73.154 ± 4.22 CG:73.929 ± 3.73	Sarcopenia	RT	RT:30 min (5 min warm-up,20 min RT and 5 min cool-down); Movements: Shoulder external rotation, elbow extension, elbow flexion, leg squat abduction, lunge and bend, shoulder abduction, half-squat stand-up	60–70% 1-RM	3(12)	vibration training	IGF-1
Kim et al. (2015) (50)	ETG:33 CG:32	ETG:81.0 ± 2.6 CG:81.0 ± 2.8	Frail older adult	AT+RT	AT: Each training session lasts 20 min, mainly including balance and gait exercises. RT: Each training session lasts 30 min, mainly using Thera-band for progressive side leg lifts, hip flexion, etc.	Borg:12–14	3(12)	Nutritional supplementation	IGF-1
Wang et al. (2019) (51)	AT:20 RT:20 AT+RT:20 CG:20	AT:64.2 ± 3.0 RT:65.1 ± 3.4 AT+RT:63.6 ± 5.2 CG:64.1 ± 2.8	Sarcopenia	AT; RT; AT+RT	RT: The older adult PRT designed by the American College of Sports Medicine (ACSM) was adopted, covering 8–10 major muscle groups (hands, feet, abdomen, pelvis, back muscles). The training starts with large muscle groups and then proceeds to small muscle groups, with 10–15 repetitions of lifting, totaling 3–5 sets. AT: The moderate-intensity aerobic exercise course designed by the American Heart Association (AHA) was used. Before exercise, there is a 5-min stretching warm-up, followed by 20 min of dynamic aerobic rhythm exercises, including in-place stepping, knee lifting, leg lifting running, arm swinging, arm raising, diamond step, and step jumping, etc., and a 5-min relaxation exercise. AT + RT: First, perform PRT for 10 min, then conduct AT for 20 min.	RT:35sets/10–15reps; AT:40–60%, VO2max; RT + AT: RT for 10 min, AT for 20 min	2(8)	Non-exercise	IGF-1
Souzu et al. (2022) (52)	ETG:14 CG:14	ETG:74.64 ± 7.13 CG:77.42 ± 6.25	Sarcopenia	RT	RT: The training selects eight major muscle groups (including chest press, leg press, vertical pull, sit-up, leg extension, arm curl, leg curl, and arm extension), with alternating training of upper and lower limbs.	50–75% 1-RM	3(12)	Nutritional supplementation	IGF-1
Chen et al. (2017) (53)	AT:15 RT:15 AT+RT:15 CG:15	AT:69.3 ± 3.0 RT:68.9 ± 4.4 AT+RT:68.5 ± 2.7 CG:68.6 ± 3.1	Sarcopenia	AT; RT; AT+RT	RT: Movements such as shoulder press, biceps curl, triceps curl, flat bench press, deadlift, leg swing, squat, standing row, single-arm row, and split squat. AT: Training movements including in-place stepping, knee lift, high knee running, rowing-style arm swing, arm swing, torso twist step, arm lift, squat, V-step, mambo step, diamond step, and step tap jump.	RT: 3 sets/8–12 reps. AT: moderate intensity (>3 metabolic equivalents)	RT: 2(8); AT: 2(8); AT+RT: 1(8)	Non-exercise	IGF-1

ETG: exercise training group;CG: control group;AT: Aerobic Training;RT: Resistant Training;AT+RT: Combination training of aerobic and anaerobic training; Borg: Borg Rating of Perceived Exertion (RPE) Scale; 1-RM:1-Repetition Maximum; HRR: Heart Rate Reserve; VO2max: Maximal Oxygen Uptake;IGF-1:insulin-like growth factor 1.

Among the included articles, 9 articles (14 RCTs) involved older adult subjects with sarcopenia (43, 45–49, 51–53), and 2 articles (2 RCTs) involved frail older adult subjects (44, 50). 5 articles (5 RCTs) adopted the exercise intervention method of combined aerobic training and resistance training (43, 46, 50, 51, 53), 7 articles (9 RCTs) used resistance training as the exercise intervention method (44, 45, 47, 48, 51–53), and 2 articles (2 RCTs) used aerobic training as the exercise intervention method (51, 53). All studies took IGF-1 as the outcome indicator.

### 3.3 Risk of bias

**Figure 2:**
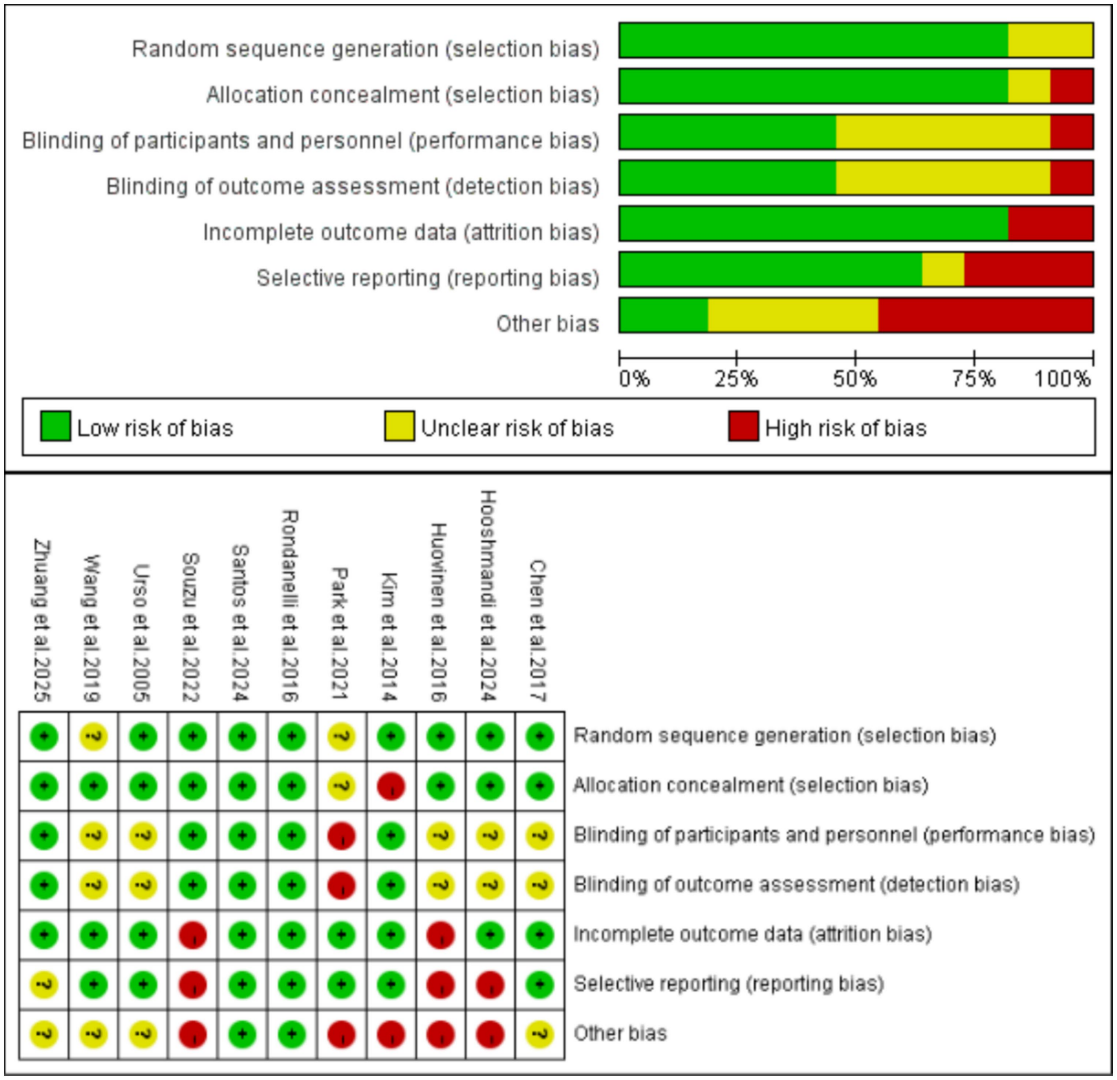
Risk of bias summary for included studies.

The risk of bias assessment results for the included studies are presented in Figure 2. Among them, 2 studies was rated as Grade A and 9 studies were rated as Grade B, indicating an overall good quality of the articles. Among the included studies, nine studies used random sequence generation (including but not limited to computer-generated random numbers, random number tables, etc.) (43–45, 47–50, 52, 53), while two studies did not clearly report this (46, 51). Nine studies provided detailed descriptions of allocation concealment methods (e.g., computer-generated random allocation sequences with concealment via opaque sealed envelopes) (43–45, 47–49, 51–53), with 1 failing to specify this information (50). Five studies reported their blinding methods (43, 48–50, 52), 5 did not clearly state this (44, 45, 47, 51, 53), and 1 indicated no blinding implementation, being judged to have a high risk of performance bias (46). Additionally, five studies reported that outcome assessors were blinded (43, 48–50, 52), 5 did not clearly state this (44, 45, 47, 51, 53), and 1 indicated no assessor blinding, being judged to have a high risk of detection bias (46). Eight studies described complete outcome data (including measurement results, dropout and attrition rates, etc.) (43, 44, 46, 48–52), whereas 3 lacked reports on dropout and attrition rates (45, 47, 52). Seven studies reported trial registration (low risk of selective reporting bias) (43, 44, 46, 48, 50, 51, 53), while 3 did not register trials and were judged to have a high risk of selective reporting bias (45, 47, 52). Based on analysis of the first five core indicators, other bias risks of the 12 studies were assessed using the “barrel shortcoming effect” principle: only 2 were low risk (43, 48), 4 unclear risk (44, 49, 51, 53), and 5 high risk (45–47, 50, 52).

### 3.4 Meta-analysis results

**Figure 3:**
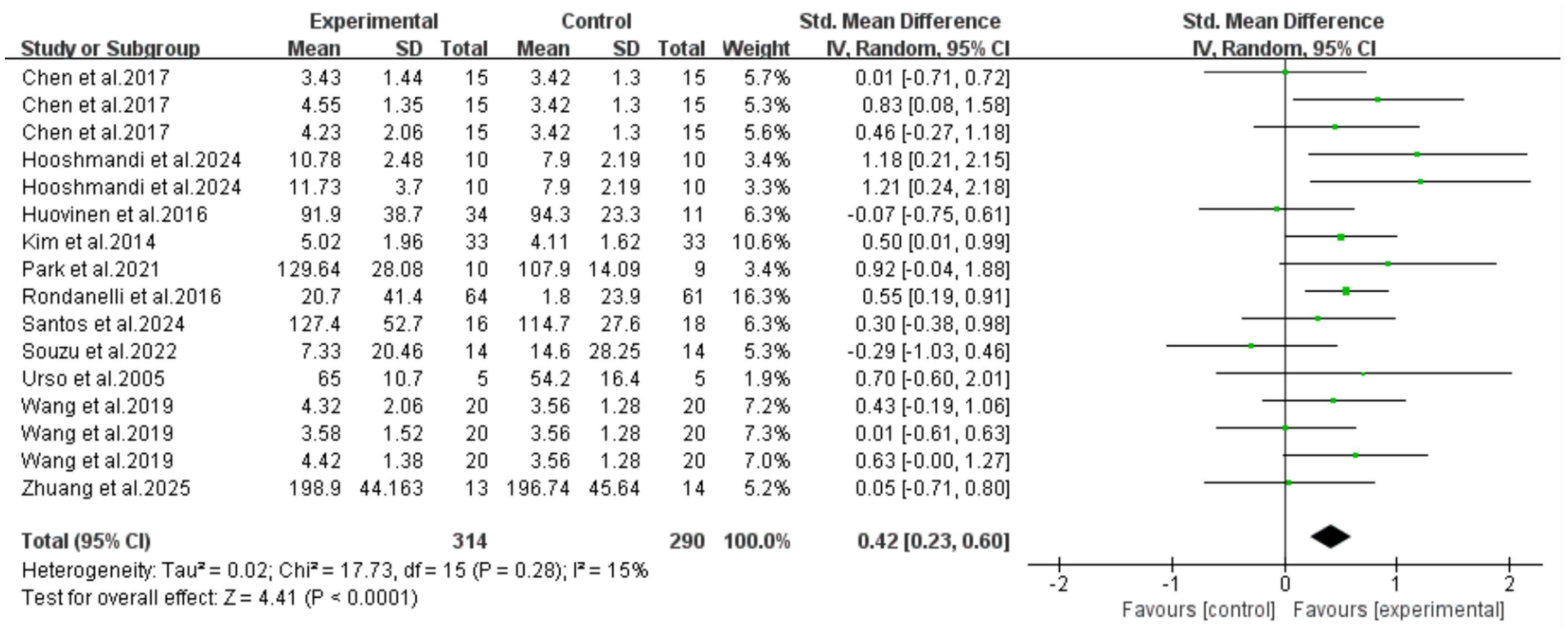
Analysis of the effect of exercise intervention on IGF-1 in older adults with frailty or/and sarcopenia.

All 11 included articles (including 16 RCTs) adopted exercise as the intervention measure to explore its intervention effect on the serum IGF-1 content in older adults with frailty and/or sarcopenia. Through Meta-analysis (Figure 3), the effect size was obtained as follows: SMD = 0.42, 95% CI: 0.23–0.60, *p* < 0.0001. The difference was statistically significant, indicating that exercise intervention can significantly increase the serum IGF-1 concentration in older adults with frailty and/or sarcopenia.

**Figure 4:**
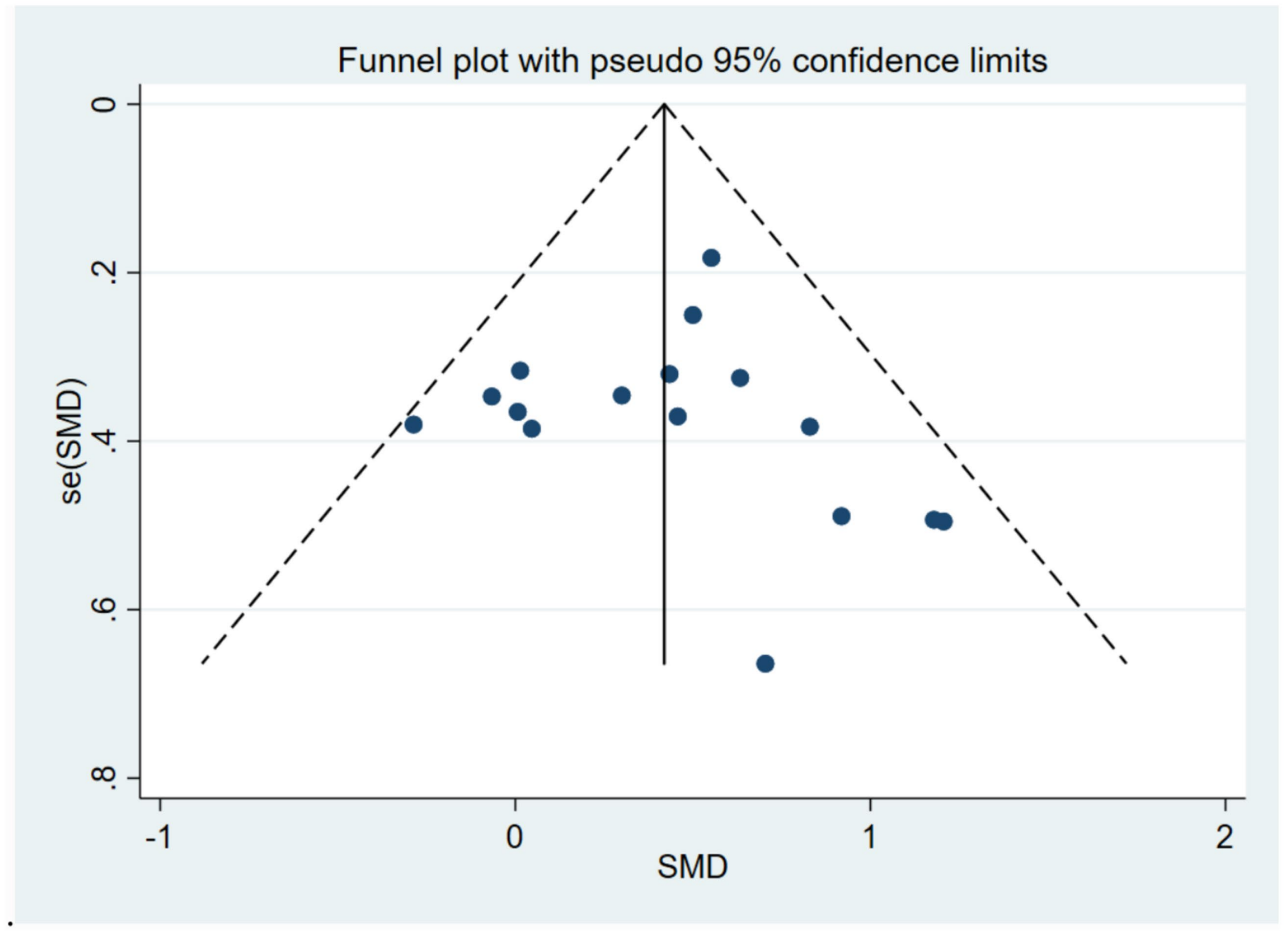
Funnel plot analysis.

The results of the heterogeneity test showed that there was moderate heterogeneity among the studies (*I*^2^ = 15%, *p* = 0.28). The Egger’s test was used to evaluate the publication bias of the 16 included studies. The results showed that *p* = 0.700, the results indicated the absence of publication bias, suggesting that the conclusions of this study have good reliability. To further verify publication bias, a funnel plot was generated for visual assessment as Egger’s test showed no significant bias (*p* = 0.700) (Figure 4). Results indicated approximately symmetric data point distribution; however, the trim-and-fill method was still needed to examine potential small-sample study missing impacts on effect sizes.

**Figure 5:**
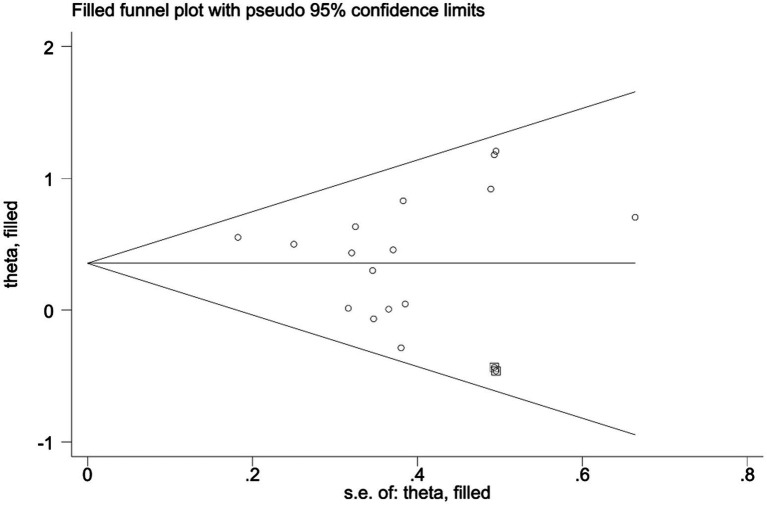
Application results of trim-and-fill method (including 11 included studies and 2 filled Studies).

After imputing two studies using the trim-and-fill method (Figure 5), the pooled effect size was slightly adjusted from SMD = 0.42 (95%CI: 0.23–0.60, *p* < 0.0001) to SMD = 0.355 (95%CI: 0.158–0.553, *p* < 0.0001). The confidence interval still did not include zero and maintained statistical significance, indicating that the core conclusions are robust.

**Figure 6:**
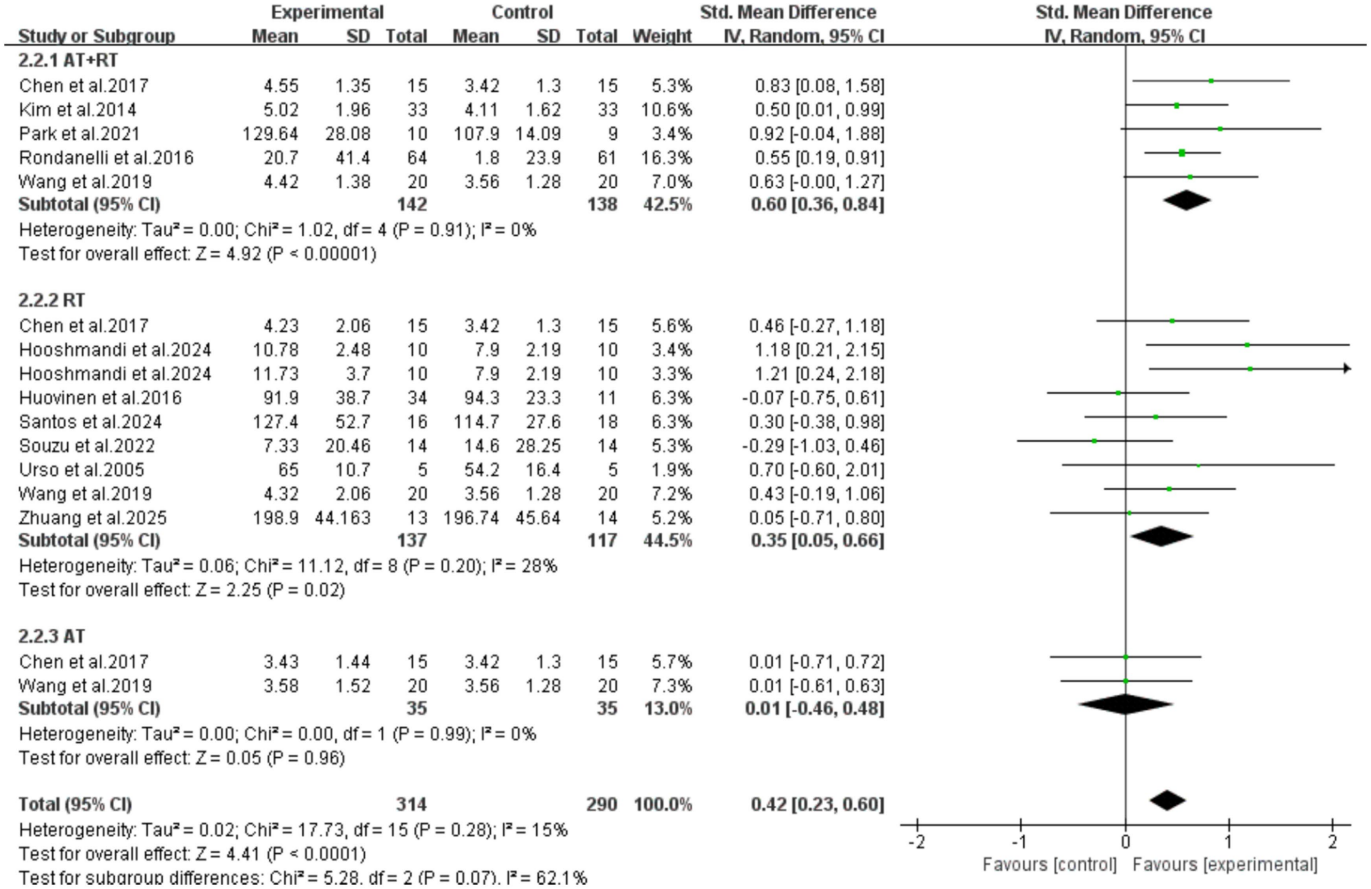
Results of subgroup analysis (different exercise intervention types).

Further subgroup analysis results showed that the intervention effect of combined aerobic training and resistance training was the best (SMD = 0.60, 95%CI: 0.36–0.84, *p* < 0.0001). Resistance training alone could also achieve an improvement with statistical significance (SMD = 0.35, 95%CI: 0.05–0.66, *p* = 0.02). However, aerobic training alone could not statistically significant increase the serum IGF-1 concentration in older adults with frailty and/or sarcopenia [SMD = 0.01, 95%CI: (−0.46, 0.48), *p* = 0.96] (Figure 6).

**Figure 7:**
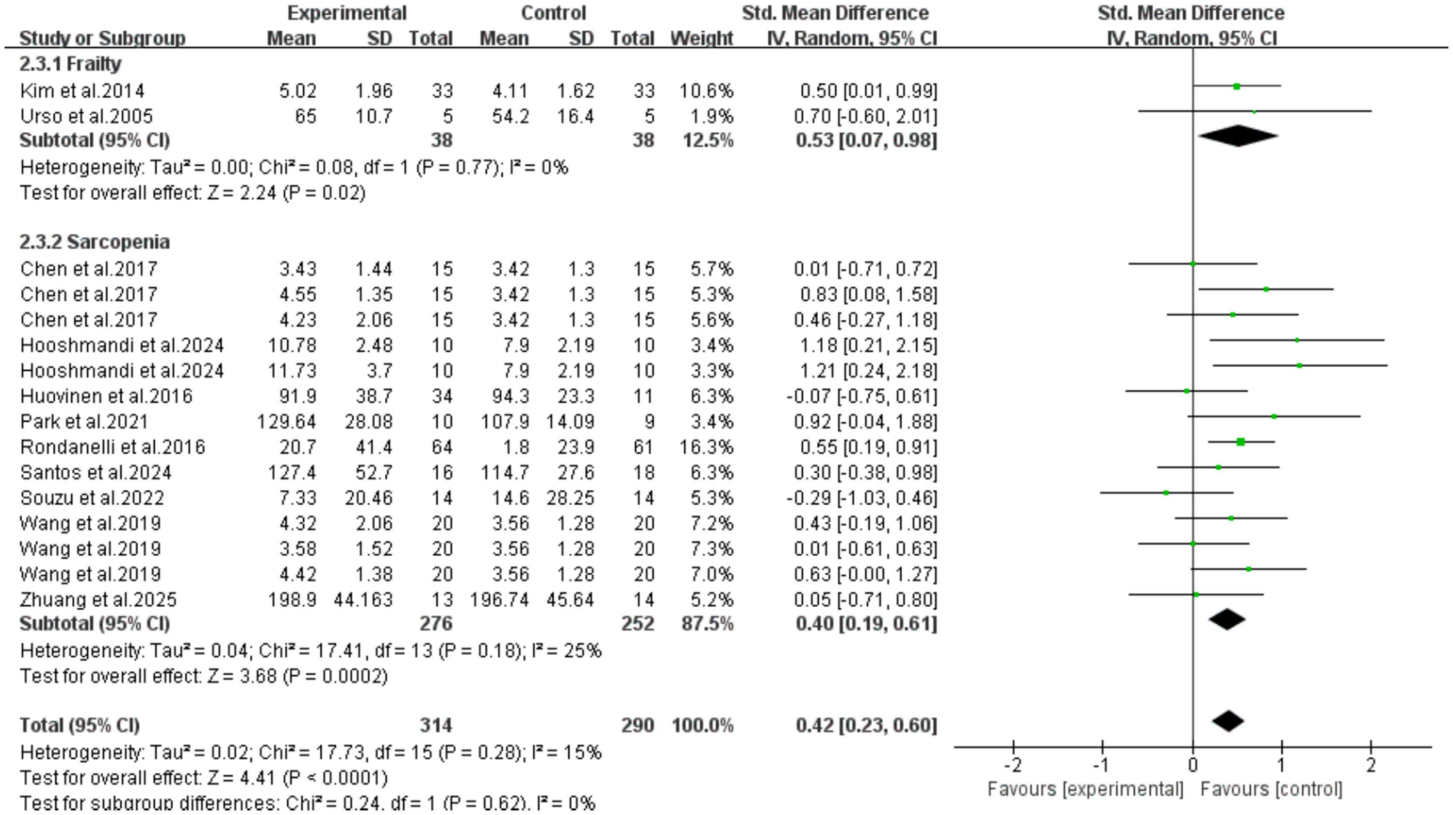
Results of subgroup analysis (subject characteristics).

Subgroup analysis was performed based on participant characteristics (frailty/sarcopenia). The results showed that exercise intervention could effectively increase serum IGF-1 concentrations in older adult individuals with frailty (SMD = 0.53, 95%CI: 0.07–0.98, *p* = 0.02) and those with sarcopenia (SMD = 0.40, 95%CI: 0.19–0.61, *p* = 0.0002). Meanwhile, there was no significant difference between the two groups (*p* = 0.62), indicating that there was no statistical difference in the improvement effect of exercise intervention on these two conditions. However, it should be noted that the number of frail patients included in this subgroup analysis was small (2 RCTs), and the extrapolation of its conclusions may be limited in clinical application. More large-sample studies are needed in the future for further verification (Figure 7).

Results of the meta-regression analysis showed that both subgroup analyses produced significant effects, indicating that the results of the two subgroup analyses had high robustness (Table 4).

**Table 4:** Meta-regression results.

Basis for subgroup analysis	β	SE	*t*	*p*	95%CI	
Different exercise intervention types	1.795	0.175	10.230	0.000	1.421	2.169
Subject characteristics	1.878	0.099	18.800	0.000	1.664	2.090

### 3.5 Sensitivity analysis

**Figure 8:**
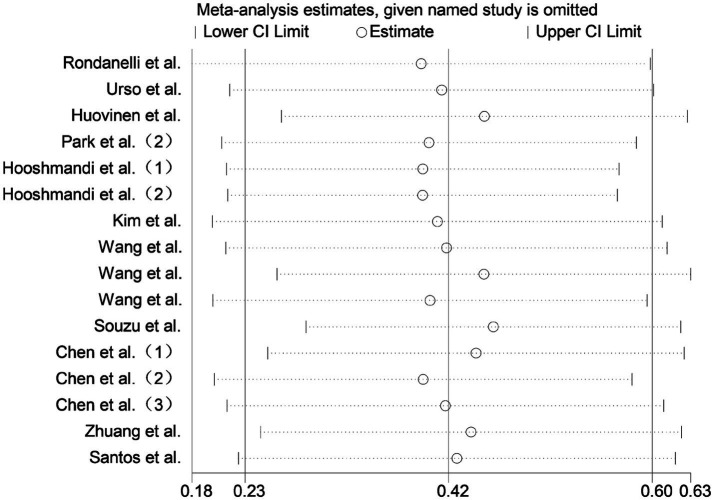
Sensitivity analysis plots for all outcomes.

Sensitivity analysis was conducted by sequentially excluding each included study. The overall effect estimates remained stable, indicating that the meta-analysis results were robust and reliable (Figure 8).

## 4 Discussion

In this study, through the Meta-analysis method, we summarized multiple RCTs on the effect of exercise intervention on serum IGF-1 concentration in older adults with frailty and/or sarcopenia. The results showed that exercise, as an intervention method, can effectively increase the serum IGF-1 concentration in patients with the above-mentioned conditions, thereby providing support for the prevention and treatment of related diseases.

This study’s results consistent with Behrad et al.’s meta-analysis, which summarized 11 RCTs and concluded that physical exercise effectively increased serum IGF-1 levels in healthy older adults (SMD = 0.276, 95%CI: 0.065–0.487) (34). Praksch et al.’s RCT further supported the beneficial effects of exercise on serum IGF-1 in the healthy older adult population (54). Our subgroup analysis demonstrated that resistance training (either alone or combined with aerobic training) exerted significantly greater effects on serum IGF-1 levels in the target population compared to aerobic training alone, which is consistent with Jiang et al.’s meta-analysis. The latter study, which pooled data from 22 RCTs on resistance training, reported a significant increase in serum IGF-1 (WMD = 10.34, 95%CI: 4.94–15.75), though the high heterogeneity (*I*^2^ = 90.3%) may relate to the broad age range of included participants (22.7–82 years) (35).

Compared with previous studies that mostly focused on healthy middle-aged and older adult people and specific intervention methods, given that older adult populations with frailty and/or sarcopenia may have pathological bases such as chronic inflammation, hormonal imbalances, and abnormal muscle metabolism, with exercise’s IGF-1 regulatory mechanisms potentially differing from those in healthy populations, this study is the first to conduct a meta-analysis targeting older adult populations with frailty and/or sarcopenia. And the research results showed that exercise may not only improve serum IGF-1 concentration in adolescents and healthy older adult people, but also may have a promoting effect on older adult populations with frailty and/or sarcopenia. Meanwhile, most previous studies adopted a single type of exercise (such as aerobic training intervention, resistance training intervention, etc.) or used mixed exercises without subdividing subgroups. For this reason, this study categorized exercises into three types of movement patterns according to the characteristics of different exercises, and directly compared their improvement effects through subgroup analyses to clarify the differential effects of different exercise types on the target population, thereby providing a basis for precise prescriptions; in addition, subgroup analysis was conducted based on the characteristics of the subjects (frailty/sarcopenia), and the results suggested that the benefits of exercise intervention for these two groups of people may be universal.

### 4.1 IGF-1 and frailty, sarcopenia

IGF-1 is a single-chain polypeptide composed of 70 amino acids, mainly synthesized and secreted by the liver, and also present in other tissues such as muscle, lung, kidney, and cartilage (55). As an important mediator of growth hormone, it plays a significant role in the development of the skeleton, muscle, and nervous system (56). The potential mechanism by which IGF-1 promotes muscle hypertrophy and alleviates the development of frailty and sarcopenia may lie in: As noted in Studies by Pedersen et al. have pointed out that IGF-1 is a myokine produced and secreted by muscle fibers (57). It can activate the IGF-1 receptor (IGF-1R) on the cell membrane, trigger and activate the downstream PI3K/Akt/mTOR and Ras/MAPK signaling pathways, promote protein synthesis in skeletal muscle cells, thereby promoting skeletal muscle hypertrophy and reducing fibrosis in muscle tissue (58). Meanwhile, the activation of PI3K/AKT by IGF-1 can also inhibit ubiquitin-mediated degradation of proteins and apoptosis in skeletal muscle cells, reduce necrosis of muscle tissue, and thus alleviate the development of sarcopenia and its comorbidities (59, 60). Specifically, specific inactivation of the IGF-1 receptor leads to a reduction in the number and diameter of muscle fibers, thereby slowing down muscle growth (61); in contrast, muscle-specific overexpression of IGF-1 results in muscle hypertrophy (62). The signal transduction of IGF-1 is initially mediated by the binding of IGF-1 ligand to IGF-1R, which induces the phosphorylation of IGF-1R dimers. The resulting phosphorylated tyrosine residues generate docking sites to recruit insulin receptor substrate 1 (IRS-1) (63). Phosphorylation of IRS-1 is required for most downstream signal transduction and represents a key step in the regulation of IGF-1 signal transduction. After IGF-1R phosphorylates IRS-1, it further activates phosphatidylinositol 3-kinase (PI3K) and its downstream protein kinase B (Akt), forming the classic IGF1R/IRS1/PI3K/Akt pathway. This is the main pathway that drives protein synthesis, inhibits protein degradation, and thereby promotes muscle cell proliferation and hypertrophy (64, 65).

It is worth noting that the concentration of IGF-1 in the human body gradually decreases with age, which can lead to growth hormone release dysfunction, decreased immune capacity, and reduced activity of the growth hormone-insulin-like growth factor axis (66). This is also one of the reasons why the older adult population is a high-risk group for frailty, sarcopenia, and their comorbidities. A study on healthy and frail older adult people found that low IGF-1 levels are associated with decreased knee extensor strength and difficulty in mobility. Additionally, other studies have shown that there is a cross-sectional relationship between IGF-1 levels and thigh muscle area and density (67). It can thus be seen that low circulating IGF-1 levels are significantly associated with sarcopenia, decreased muscle strength, and impaired physical function in the older adult (68). Meanwhile, a Japanese study also showed that in men, older adult individuals with low IGF-1 levels (≤88 ng/mL) had a 1.58-fold and 3.42-fold higher probability of developing pre-frailty and frailty, respectively, compared to those with high IGF-1 levels (≥125 ng/mL). In women, low IGF-1 levels (≤78 ng/mL) were also positively correlated with an increased risk of frailty (69).

In summary, as an important factor regulating bone and muscle metabolism, the decline in IGF-1 levels associated with aging, reduced exercise, malnutrition, and other factors may be one of the pathological bases for the development of older adult frailty and sarcopenia. This further suggests that exercise intervention to increase serum IGF-1 concentrations in this population may have certain clinical significance in the present study.

### 4.2 IGF-1 and exercise

Compared with exogenous treatments such as nutritional supplementation and drug therapy, exercise is a safer and lower-cost preventive and therapeutic method by promoting the endogenous secretion of IGF-1 in the body (70–72). A study by Gomarasca et al. found that skeletal muscle, as an organ with endocrine function, can secrete bioactive protein IGF-1 after exercise stimulation, promoting the improvement of skeletal muscle mass, thereby preventing or treating the occurrence and development of sarcopenia (73). Meanwhile, IGF-1 in skeletal muscle has autocrine and paracrine functions, among which a muscle-specific form is called mechano growth factor (MGF), whose concentration is significantly upregulated during stretching and increased load (74). A long-term resistance training study on the older adult population found that the serum IGF-1 of the subjects increased significantly, and at the same time, cognitive function and lean body mass were also improved (75). Both resistance exercise and aerobic endurance exercise can effectively activate the IGF-1/PI3K/Akt/mTOR signaling pathway, thereby increasing the levels of IGF-1, IGF-1R and mTOR, as well as the activities of PI3K and Akt (76, 77). In terms of acute exercise effects, studies have found that 10 min of high-intensity resistance training or high-intensity aerobic training can effectively promote the increase of IGF-1 concentration in the human circulation and enhance the bioavailability of IGF-1 (78).

Despite extensive evidence that exercise intervention can effectively promote an increase in circulating IGF-1 concentration in the body, some studies have drawn opposite conclusions, suggesting that exercise intervention cannot increase or even reduce IGF-1 concentration in the body (79, 80). A study by Nindl et al. explained that such differences in results may stem from the lack of dietary control, heterogeneity of subject characteristics, differences in plasma volume, variations in exercise modes, and differences in sampling time and process after exercise (81). In addition, experimental results have shown that the decrease in IGF-1 levels during high-intensity exercise may promote the secretion of GH by weakening the negative feedback effect, thereby increasing circulating GH levels and promoting fatty acids as an energy source for exercising muscles. Meanwhile, long-term exercise may also affect the recovery of IGF-1 after exercise (82).

Through subgroup analysis of different exercise intervention types, this study found that the combined training of aerobic and resistance training had a relatively better effect on improving serum IGF-1 concentration in older adult patients with frailty and/or sarcopenia, followed by resistance training alone, while the improvement effect of aerobic training did not reach statistical significance. Based on the logical review of existing studies, the potential mechanism may be as follows: resistance training, by applying mechanical stimulation to skeletal muscles, may promote the acute secretion of growth hormone (GH) and activate proteases such as ADAM10 (A Disintegrin And Metalloproteinase 10) and ADAM17 (A Disintegrin And Metalloproteinase 17) on the cell membrane (83, 84). GH is the main upstream signal promoting hepatic synthesis of IGF-1, and the activation of proteases may also create conditions for the release of IGF-1 in local tissues. Subsequent aerobic training can promote vasodilation, reduce intravascular resistance, and increase blood flow, which may improve the delivery efficiency of GH, ADAM10, ADAM17 and other proteases generated or activated by resistance training to the liver and related target tissues (85). Therefore, a reasonable hypothesis is that resistance training can promote GH secretion and activate ADAMs activity through generating local mechanical stress, while aerobic training further accelerates the above process by improving blood circulation. The combination of the two training methods may result in a higher efficiency of IGF-1 secretion than a single training method.

Results of this subgroup analysis showed that combined aerobic and resistance training had a better effect on improving serum IGF-1 concentration in older adults with frailty and/or sarcopenia than single training methods, which provides a reference basis for clinically formulating targeted exercise prescriptions. Meanwhile, the hypotheses formed by deducing the potential mechanisms in the subgroup analysis results based on existing theories may provide certain research directions for future clinical trials targeting frailty and sarcopenia. In addition, the research results further suggest the potential of exercise intervention in increasing serum IGF-1 concentration in specific populations, providing support for non-pharmacological treatment of sarcopenia and frailty.

## 5 Strengths and limitations

The innovations and advantages of this study are mainly reflected in the following aspects: First, by precisely focusing on the research gaps in the field, it is the first to target the high-risk population of older adult patients with frailty and/or sarcopenia, and specifically explore the intervention effect of exercise intervention on their serum IGF-1 concentration, which helps to address the lack of population specificity in existing meta-analyses. Second, the study strictly followed the PRISMA guidelines and completed the pre-registration of the research protocol on the PROSPERO platform (registration number: CRD420251071083), which is conducive to ensuring the transparency and reproducibility of the research process. In addition, subgroup analysis showed that resistance training and combined training with resistance training had relatively better improvement effects on outcome indicators, providing a relatively intuitive basis for selecting exercise types when formulating targeted exercise prescriptions.

However, at the same time, this study still has the following limitations: (1) Limitations in the quality of original articles. The overall quality of the included studies is limited, with only 2 study being of Grade A and the rest being Grade B. Some studies did not implement blinding and allocation concealment, which may lead to a certain degree of selection bias and performance bias. (2) Constraints from sample size and population heterogeneity. Although systematic searches have been conducted in major databases, there are few articles that meet the inclusion criteria. This study only included 614 subjects (including 16 RCTs), and both the frailty subgroup and the aerobic training subgroup included only 2 studies. Meanwhile, the types of sarcopenia-related comorbidities (such as sarcopenic osteoporosis, sarcopenic obesity, etc.) were not subdivided, which may result in insufficient consideration of disease-specific manifestations. (3) Insufficient standardization of exercise intervention parameters. The included studies have large heterogeneity in intervention cycle, duration, intensity and frequency, making it difficult to extract the optimal dose-effect relationship. (4) Lack of analysis on long-term effects. The intervention cycles of the included RCTs are all ≤16 weeks, so the long-term intervention effect of exercise on IGF-1 cannot be evaluated.

In view of the existing limitations of this study, future research can be deepened and improved from the following dimensions: (1) Further strengthen the research on targeted exercise prescriptions, standardize the specific parameters of exercise intervention, further explore its dose-effect relationship, and clarify the optimal intervention parameter matrix; at the same time, combine new training modes such as blood flow restriction training and high-intensity interval training to enrich the range of exercise intervention options. (2) Expand the clinical trial cycle and carry out exercise intervention experiments with a duration of ≥48 weeks to evaluate the long-term effect of exercise intervention and its role in delaying disease progression. (3) Explore combined intervention strategies, carry out intervention studies on the combination of exercise with nutrition and drugs, and clarify the impact of various nutritional interventions on the effect of exercise intervention, as well as the role of exercise in reducing drug side effects. (4) Strengthen the evidence base for special populations, carry out multi-left, large-sample RCTs to improve the credibility of research evidence; at the same time, incorporate economic cost-effectiveness into the analysis to provide more references for the selection of disease prevention and treatment methods and the formulation of public health policies.

## 6 Conclusion

This study employed the Meta-analysis method to systematically integrate and quantitatively synthesize existing relevant studies. The results showed that exercise intervention can help increase the serum IGF-1 concentration in older adults with frailty and/or sarcopenia. Meanwhile, further subgroup analysis clarified that resistance training and combined training with resistance training are more effective than aerobic training alone in increasing serum IGF-1 in such patients. Therefore, to enhance clinical practicability, it is proposed that a combined resistance and aerobic training program could be adopted under safety supervision. The specific program settings are as follows: the training period is recommended to be ≥12 weeks, with 3–5 training sessions per week; the intensity of resistance training is suggested to be set at 60–80% 1-RM, and the intensity of aerobic training is moderate (i.e., Borg scale 12–14 or 40–60% of VO₂max). The total duration of each intervention session is 50–60 min, with approximately 25–30 min allocated to resistance training and 25–30 min to aerobic training, respectively. This evidence-based prescription may serve as a basic intervention module in community rehabilitation and geriatrics departments, helping delay muscle loss by regulating the IGF-1 pathway and laying a practical foundation for exercise intervention strategies. In the context where current exogenous treatment methods have certain limitations, this study provides preliminary evidence for the prevention and treatment of older adult frailty and sarcopenia, and may have certain guiding significance for clinical practice.

## Data Availability

The original contributions presented in the study are included in the article/Supplementary material, further inquiries can be directed to the corresponding authors.

## Footnotes

^1^ https://www.crd.york.ac.uk/PROSPERO/view/CRD420251085472
