# Supplementary material for: Exercise interventions and serum IGF-1 levels in older adults with frailty and/or sarcopenia: a systematic review and meta analysis
Source: Front Public Health. 2025 Aug 21;13:1660694. doi: 10.3389/fpubh.2025.1660694 (PMC12408309; doi:10.3389/fpubh.2025.1660694)
Supplement: Supplementary file 1 [file Table_1.DOCX]

### ****Literature Search Strategy Report​**​**

****For Systematic Review & Meta-Analysis:​****​
Exercise Interventions and Serum IGF-1 Levels in Older Adults with Frailty and/or Sarcopenia

#### **​**​1. Study Objective​**​**

To systematically identify randomized controlled trials (RCTs) evaluating the effects of exercise interventions on serum insulin-like growth factor-1 (IGF-1) levels in older adults (≥60 years) diagnosed with frailty and/or sarcopenia.

#### **​**​2. Data Sources​**​**

​****​English Databases:​****​

PubMed, Web of Science , Cochrane Library , EMBASE , Scopus , Google Scholar (supplementary search), CNKI

​****​Timeframe:​****​
From database inception to ****July 20, 2025​****​

#### **​**​3. Search Strategy Development​**​**

​****​Conceptual Framework:​****​
Three core concept groups were combined using Boolean operators:

| Concept groups | Search terms |
| --- | --- |
| Insulin Like Growth Factor-1 | Insulin-Like Growth Factor I, IGF-1, Insulin Like Growth Factor I, Somatomedin C |
| Exercise Intervention | Exercise, Physical Exercise, Aerobic Exercise, Blood Flow Restriction Therapy, High-Intensity Interval Training, Resistance Training, Endurance Training |
| Target population | senior citizen, Aged, Elderly, Frailty, Sarcopenia, Frailties, Frailness，Frailty Syndrome, Sarcopenias |
| Research type | Randomized controlled trial |

​****​Search Syntax :​****​

#1 "insulin like growth factor i"[MeSH Terms] OR "IGF-1"[Title/Abstract] OR "IGF-I"[Title/Abstract] OR "IGF-I-SmC"[Title/Abstract] OR "insulin like growth factor i"[Title/Abstract] OR "insulin like somatomedin peptide i"[Title/Abstract] OR "insulin like somatomedin peptide i"[Title/Abstract] OR "somatomedin c"[Title/Abstract]

#2 "Exercise"[MeSH Terms] OR "Exercises"[Title/Abstract] OR "exercise physical"[Title/Abstract] OR "exercises physical"[Title/Abstract] OR "physical exercise"[Title/Abstract] OR "physical exercises"[Title/Abstract] OR "exercise aerobic"[Title/Abstract] OR "aerobic exercise"[Title/Abstract] OR "aerobic exercises"[Title/Abstract] OR "exercises aerobic"[Title/Abstract] OR "exercise isometric"[Title/Abstract] OR "exercises isometric"[Title/Abstract] OR "isometric exercises"[Title/Abstract] OR "isometric exercise"[Title/Abstract] OR "acute exercise"[Title/Abstract] OR "acute exercises"[Title/Abstract] OR "exercise acute"[Title/Abstract] OR "exercises acute"[Title/Abstract] OR "exercise training"[Title/Abstract] OR "exercise trainings"[Title/Abstract] OR "training exercise"[Title/Abstract] OR (("education"[MeSH Subheading] OR "education"[All Fields] OR "Training"[All Fields] OR "education"[MeSH Terms] OR "train"[All Fields] OR "train s"[All Fields] OR "trained"[All Fields] OR "training s"[All Fields] OR "Trainings"[All Fields] OR "trains"[All Fields]) AND "Exercise"[Title/Abstract]) OR "physical activity"[Title/Abstract] OR "activities physical"[Title/Abstract] OR "activity physical"[Title/Abstract] OR "physical activities"[Title/Abstract] OR "blood flow restriction therapy"[Title/Abstract] OR "high intensity interval training"[Title/Abstract] OR "resistance training"[Title/Abstract] OR "endurance training"[Title/Abstract]

#3 "Aged"[MeSH Terms] OR "Aged"[Title/Abstract] OR "Elderly"[Title/Abstract] OR "senior citizen"[Title/Abstract]

#4 "Frailty"[MeSH Terms] OR "Frailties"[Title/Abstract] OR "Frailness"[Title/Abstract] OR "frailty syndrome"[Title/Abstract] OR "Debility"[Title/Abstract] OR "Debilities"[Title/Abstract]

#5 "sarcopenia"[MeSH Terms] OR "Sarcopenias"[Title/Abstract]

#6 ("randomized controlled trial"[Publication Type] OR "randomized controlled trials as topic"[MeSH Terms] OR "randomized controlled trial"[All Fields] OR "randomised controlled trial"[All Fields]) AND (meta-analysis[Filter])

#7 #4 OR # 5

#8 #1 AND #2 AND #3 AND #6 AND #7

#### **​**​4. Screening Process​**​**

​****​Phase 1: Deduplication​****​

Used EndNote X9 to remove duplicates

​****​Phase 2: Title/Abstract Screening​****​

Two independent reviewers (MML & YND) applied ​****PICOS criteria****:

1)Age ≥60 with frailty/sarcopenia diagnosis

2)Intervention measures: The experimental group receives intervention through single or multiple exercise methods such as comprehensive training, aerobic training, and resistance training;

3)Control measures: The control group adopts measures such as health education, routine nursing, and routine exercise, which are different from the experimental group in terms of intervention methods or intervention objects;

4)Study type: Only randomized controlled trials (RCTs) are included, regardless of whether blinding is used;

5)Outcome indicators: The outcome indicators involved in the study should include IGF-1.

​****​Phase 3: Full-Text Review​****​

Discrepancies resolved through consensus (arbitrated by corresponding author Yeshou Xie)

#### **​**​5. Search Results​**​**

| **Stage** | **Records** | **Action Taken** |
| --- | --- | --- |
| Initial Search | 874 | Across 6 databases |
| After Deduplication | 513 | 361 duplicates removed |
| Title/Abstract Screening | 67 | 446 irrelevant studies excluded |
| Final Included Studies | 12 (17 RCTs) | Some studies had multiple arms |

#### ****6. Quality Control Measures​**​**

Protocol Registration: PRISMA-compliant protocol on PROSPERO (ID: CRD420251085472)

Dual Independent Review: Screening, data extraction, and RoB2 assessment

Database Coverage: 6 major biomedical databases + Google Scholar

Bias Assessment:

Egger's test: p=0.700 (no significant publication bias)

#### **​**​7. Limitations​**​**

Grey Literature: Unpublished conference abstracts/theses not systematically searched

Cut-off Date: Studies after July 2025 not captured

#### **​**​8. Validation Methods​**​**

Sensitivity Analysis: Sequential exclusion of individual studies (Figure 7)

Alternative Search Strategies: Tested variations in MeSH/keyword combinations
